# Enzymatic Polymer Brush Interfaces for Electrochemical Sensing in Biofluids

**DOI:** 10.1021/acsabm.5c00146

**Published:** 2025-04-24

**Authors:** Jesper Medin, Maria Kyriakidou, Bagus Santoso, Pankaj Gupta, Julia Järlebark, Andreas Schaefer, Gustav Ferrand-Drake del Castillo, Ann-Sofie Cans, Andreas Dahlin

**Affiliations:** † Department of Chemistry and Chemical Engineering, Chalmers University of Technology, 41296 Gothenburg, Sweden; ‡ Nyctea Technologies AB, AstraZeneca BioVentureHub, 431 83 Mölndal, Sweden

**Keywords:** neurotransmitters, electrochemistry, biosensors, antifouling, polymer brushes, enzymes, cascade reactions

## Abstract

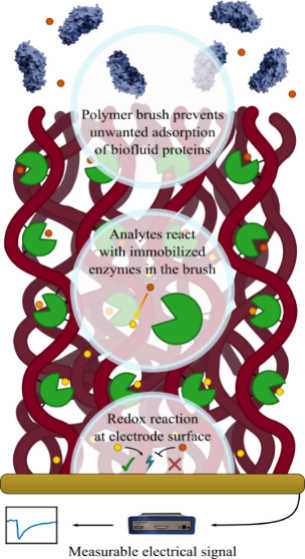

Electrochemical sensors enable specific and sensitive detection of biological markers. However, most small molecule analytes are not electroactive. Therefore, enzymes are widely used for selective breakdown of the markers into electro-active species. However, it has proven difficult to design a sensor interface where any enzyme can be controllably immobilized in high amounts with preserved activity. In addition, most interfaces cease to function in biofluids due to “fouling” of the sensor surface. Here we present a generic strategy employting polymer brushes for enzymatic electrochemical sensing which resolves these issues. Generic conjugation chemistry is used to covalently bind large amounts of enzymes (>1 μg/cm^2^). Remarkably, despite this enzyme load, the (∼200 nm thick) brushes remain highly hydrated and practically invisible by electrochemical methods: Small molecules freely access the underlying electrode and the charge transfer resistance increment is exceptionally low (<10 Ω). The enzymatic polymer brush interfaces enable specific detection of the biomarkers glucose and glutamate by simple chronoamperometry. Furthermore, by sequential immobilization of several enzymes, cascade reactions can be performed, as illustrated by detection of acetylcholine. Finally, the sensor interface still functions in cerebrospinal fluid (10× diluted, unfiltered). In conclusion, polymer brushes provide extended possibilities for enzymatic catalysis and electrochemical sensing.

## Introduction

Electrochemical biosensors are widely researched for monitoring health conditions and for diagnostic applications in detecting biomarkers at early stages of disease development due to their high sensitivity and selectivity.^1^ This is because of their ability to rapidly and continuously monitor electron transfer events, enabling quantification of electroactive species present at the electrode surface. Hence, the sensors provide direct monitoring of the release of electroactive biomarkers at biologically relevant time and length scales. These are important features for the development of diagnostic sensors for brain-related disorders, where neurotransmitters can serve as biomarkers for conditions such as depression and Alzheimer′s disease.^2,3^ Most importantly, amperometric measurements offer detection of neurotransmitter biomarkers for neurological pathologies, with submillisecond temporal resolution^4,5^ and spatial resolution down to the single micrometer scale.^6^ For instance, the placement of enzyme-based microelectrode biosensors in the brain allows the long-term recordings of neurochemical activity in response to behavior in both healthy and diseased animals.^7,8^ Additionally, pioneering work by Hochstetler et al.^9^ showed that positioning this type of microsensors in rodent brain tissue slices can provide submillisecond temporal resolution of single exocytotic neurotransmitter release on the level of individual neurons. This high temporal resolution has made it possible to study the kinetics of single synaptic vesicle fusion pores regulating the amount of neurotransmitters released into synapses,^10,11^ which provides important information on how neurons control signaling strength during neuronal communication.

Whereas some neurotransmitters that are relevant biomarkers, such as dopamine, are naturally electroactive, several of the major neurotransmitters in the brain are not. This includes key neurotransmitters associated with neurodegenerative diseases, such as glutamate and acetylcholine in Alzheimer’s disease,^12^ which makes electrochemical detection impossible unless the marker can be converted from a nonelectroactive state to one capable of undergoing redox reactions at the electrode surface. This can be solved by immobilization of chemically selective enzymes capable of breaking down these neurotransmitters into species that are electrochemically detectable.^13^ As an added benefit, enzymes also provide chemical selectivity for a single substrate, making enzyme-modified electrodes attractive for sensing in complex chemical environment and in vivo.^14^

However, electrodes with enzymes used for electrochemical biosensing are highly susceptible to fouling from the complex biological environment. Biofouling, i.e. nonspecific adsorption of biological molecules, will severely reduce electrode lifetime and sensitivity.^15^ Also, direct adsorption of enzymes onto solid surfaces tends to reduce their activity.^16^ This calls for the use of tethers of soft matter constructs on the surface for a more gentle enzyme immobilization^17^ and a proper quantification of immobilized amount as well as specific activity, to be compared with the free enzyme in solution.^18,19^ Unfortunately it has proven difficult to achieve such constructs while also maintaining a high redox-activity, i.e. it is difficult to keep the electrode accessible for efficient charge transfer events.^20^ In addition, if the coatings are thicker than ∼1 μm there are strongly detrimental effects on the response time and the ability to detect transient signals.^21^ In this context, polymer brushes, i.e. end-grafted chains at high surface coverage, are an interesting surface functionalization strategy.^22^ Still, work to date has been done on brushes with the model enzyme glucose oxidase^23−26^ (GOx) and detection in biofluids has not been demonstrated.

Here we present a generic strategy for enzymatic electrochemical detection in biofluids based on poly(acrylic acid) (PAA) brushes. The PAA brushes, which are here around 200 nm in their hydrated state, can be used to conjugate enzymes in a generic manner, at high capacity (3D instead of 2D) and with largely preserved activity. Remarkably, we show that by using appropriate chemistry for grafting, polymerization and bioconjugation, the highly hydrated enzymatic brushes become almost impossible to detect electrochemically: their presence is not noticeable in cyclic voltammetry (CV) and barely detected in electrochemical impedance spectroscopy (EIS), even though the enzyme amount on the surface is extremely high. As a proof-of-principle for enzyme-mediated detection we use amperometry to detect various analytes important to the brain, such as glucose and glutamate, with the corresponding oxidative enzymes immobilized in the polymer brush matrix. In addition, we show that a cascade reaction dependent on multiple enzymes, the catalytic breakdown of the neurotransmitter acetylcholine, can be achieved inside the brush by subsequent immobilization of the different sequential enzymes. Finally, neurotransmitters can also be detected in cerebrospinal fluid, showing that the polymer brushes prevent severe fouling of the surface. To the best of our knowledge, this is the first study to combine polymer brushes with enzyme-mediated electrochemical detection of neurotransmitters. Additionally, our work provides the first example of a functional polymer brush interface with electrochemical redox activity comparable to that of an unmodified electrode. The results hold significant potential for the development of electrochemical sensors and other applications involving polymer brushes.

## Results and Discussion

### Enzyme Immobilization and Quantification

**Figure 1:**
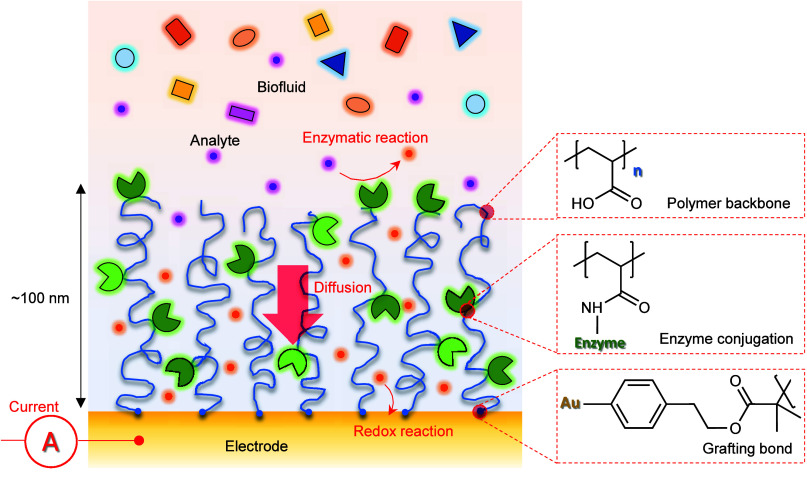
Illustration of the interface design. Poly(acrylic acid) brushes are functionalized with enzymes by linking −COOH and −NH_2_ groups. The polymers are securely anchored by sparse aryl bonds so that the electrode remains highly accessible for Faradaic reactions.

PAA brushes were prepared on gold electrodes using diazonium salt grafting (Figure 1) and atom transfer radical polymerization (ATRP) as described previously.^27^ In previous work, we have also shown that PAA as well as poly(methacrylic acid) brushes in their protonated state exhibit generic attractive interactions with water-soluble proteins.^17^ We and others^28^ have attributed this effect to hydrogen bonds, similar to the well-known phenomenon of polymeric carboxylic acids in solution forming complexes with other hydrophilic polymers.^29^ As the brush is much thicker than the characteristic size of proteins, they can bind in multilayers inside the brush. For secure enzyme immobilization, covalent bonds need to be formed, which can be achieved by the established EDC/NHS conjugation protocol. The initial modification with EDC/NHS creates a “clickable” NHS ester group on the polymer brush which forms an amide bond with amines on proteins.^30^ However, if the native brush does not have favorable interactions with proteins, the enzymes will not penetrate the brush interior and the immobilized amount will then be very low.^31^ Hence, we opted for conditions where the enzymes can move through the brush by hydrogen bonds (with the −COOH groups) while also being able to form covalent bonds (with the −NHS groups). To achieve this, the ionic strength was lowered compared to physiological levels, and the pH was reduced to 5.0 (10 mM MES with no added salt). Under these conditions the PAA brush is almost entirely neutral^32^ and spontaneous NHS hydrolysis should not occur too quickly.^33^ GOx was used as a model enzyme for the initial characterization and proof-of-principle measurements in this work as it is since long ago used in enzymatic biosensors.^34^

**Figure 2:**
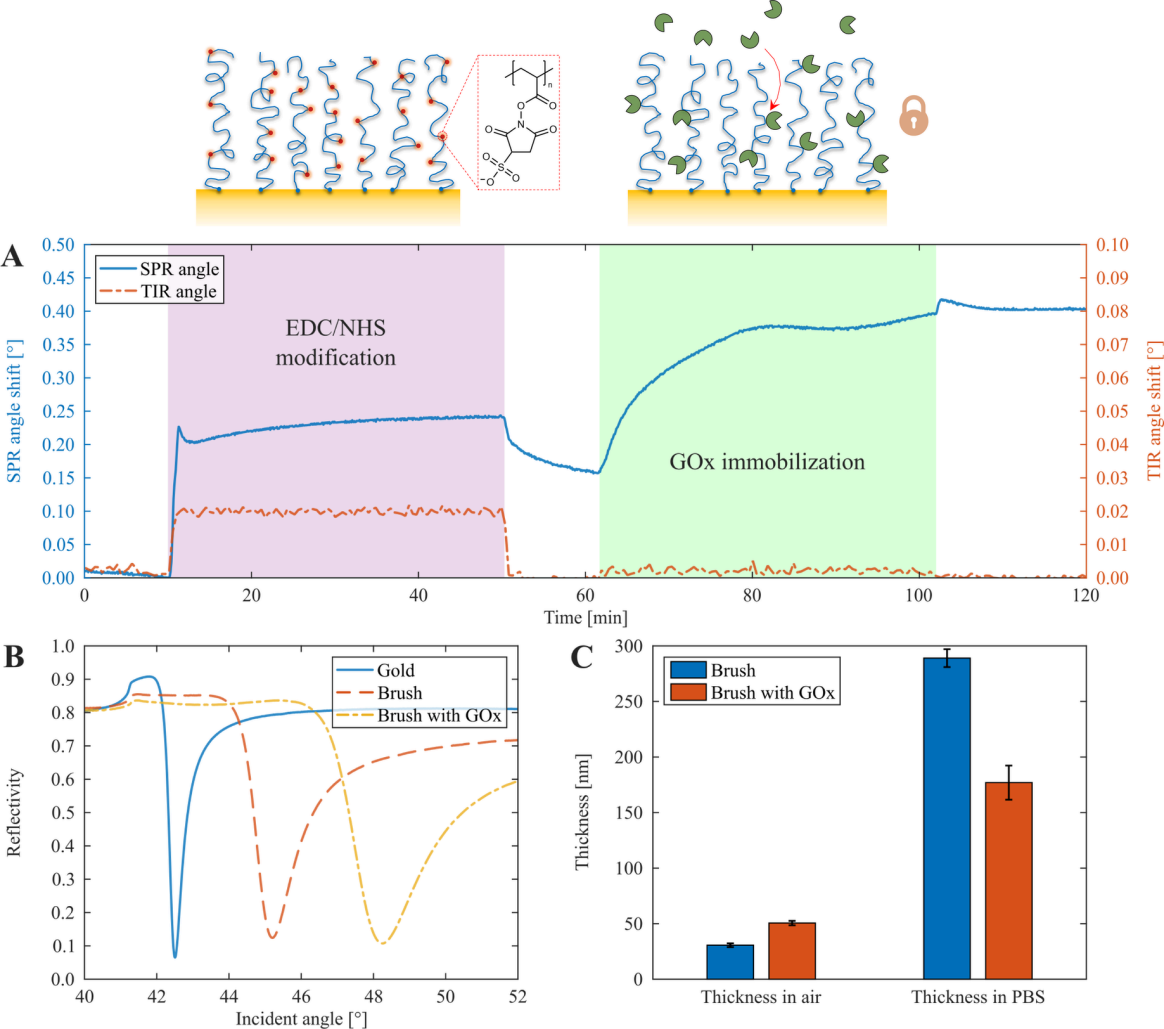
Enzyme immobilization and quantification by SPR. (A) Real-time SPR data of in situ EDC/NHS functionalization (5 mM EDC + 10 mM NHS) and enzyme conjugation (500 μg/mL). The running buffer is 10 mM MES pH 5.0. The change in total internal reflection (TIR) angle, which corresponds to the bulk refractive index, is also shown. (B) Dry spectra before and after polymerization and immobilization of GOx ex situ. Note that the experimental uncertainty is much smaller (∼0.01°) than the signals. (C) Dry thicknesses and exclusion heights in PBS for the PAA brush before and after enzyme conjugation. Error bars represent instrumental variation.

Each step of immobilization was confirmed by surface plasmon resonance (SPR) in real-time (Figure 2A). Introducing NHS groups to the polymer brush gave a response of 0.25° which increased to 0.40° after GOx binding (at 980 nm). The SPR trace also showed that the hydrolysis was not too rapid as most NHS groups remained when the enzymes were introduced 10 min later. After washing with PBS and water, the enzyme amount was quantified by spectra in the dry state^18^ (Figure 2B). Note that at physiological ionic strength any enzymes that are not covalently bound will be released again upon returning to physiological pH due to electrostatic repulsion unless they are highly positively charged^17^ (GOx has pI 4.2). Our protocol resulted in remarkably high covalent enzyme immobilization capacity, i.e., 1–2.5 μg/cm^2^ for PAA brushes with dry thickness of 20–30 nm. Considering that the dimensions of GOx (160 kg/mol) are 60 × 52 × 77 Å^3^,^35^ the average surface area occupied by a single GOx is 39.1 nm^2^ and the maximum density of a monolayer should then correspond to 680 ng/cm^2^. As this is merely a quarter of the measured GOx surface coverage (Table S1), the enzymes are clearly able to bind in multilayers within the hydrated polymer brush, confirming that they can reach deep into the brush (ternary adsorption) during the conjugation process. The uniformity of the enzymatic brush coating was also good, with 5–10% variation over ∼1 cm^2^ surfaces. The PAA brushes alone showed similar variation, suggesting that the enzyme to polymer ratio was constant over the surface. The immobilized amount could be increased even further by tuning the protocol. However, we speculated that mass coverages on the order of 1 μg/cm^2^ should be more than sufficient to create an efficient catalytic interface considering that previous work has shown that monolayers of directly adsorbed enzymes can provide fast biosensors.^36,37^ Immobilization of smaller enzyme amounts is straightforward by simply interrupting the protein binding process.

Furthermore, we used noninteracting probes in SPR to measure the height of the enzyme-functionalized polymer brushes in physiological PBS buffer (pH 7.5). In brief, injections of poly(ethylene glycol) were used to obtain a so-called exclusion height based on the SPR bulk response.^29^ An average degree of hydration of the films could then be obtained by comparing this height with the dry thickness (Figure 2C). These measurements showed that unmodified PAA brushes had a water content of over 90%, i.e. the chains were very strongly stretched, which is largely due to self-repulsion from the high degree of ionization at physiological conditions in terms of pH and salt.^32^ After enzyme immobilization the exclusion height decreased, which we attribute to multivalent interactions between proteins and multiple polymer chains.^38^ Nevertheless, the enzyme-functionalized films remained highly hydrated (∼70%) even though the enzyme amount is comparable to the polymer amount in terms of mass coverage. Together with the fact that the grafting layer with aryl bonds is very thin,^27^ this suggests that the electrode underneath the brush might be highly accessible for redox reactions.

### Electrochemical Characterization of the Interface

**Figure 3:**
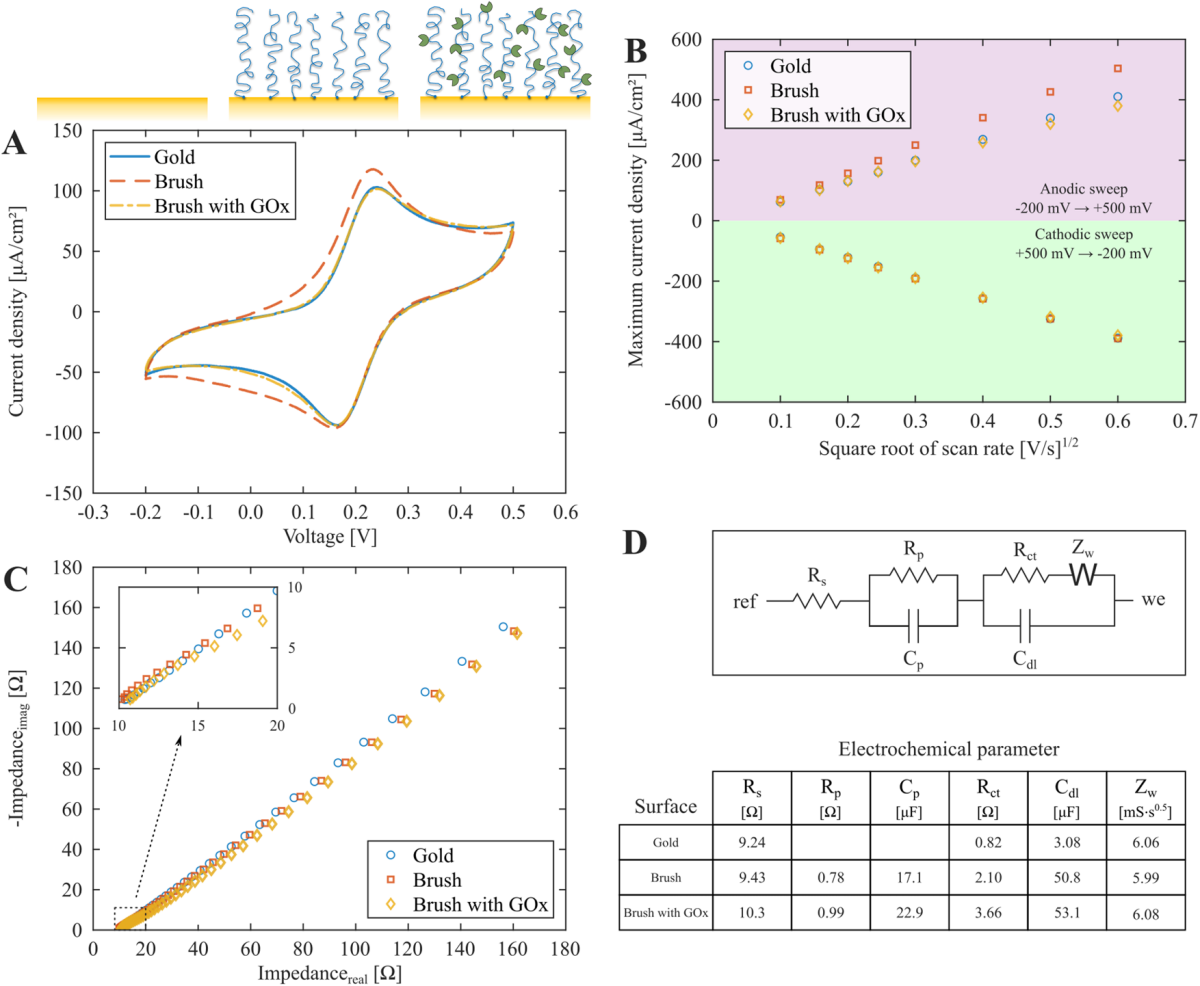
Electrochemical characterization of the sensor interface. (A) CV data (third cycle) of bare gold, after polymer brush formation and after GOx immobilization (1.26 μg/cm^2^) measured at 25 mV/s in the presence of 1 mM ferrocenemethanol. (B) Randles-Sevcik plots of peak current values vs scan rate during CV. (C) EIS spectra in Nyqvist plots measured with 0 DC bias vs the standard redox potential of the redox probe. Each spectrum is the average of three repeats (variation was comparable to the symbol size). The inset shows the higher frequency range up to 10 kHz. Electrode area 1.76 cm^2^. (D) Equivalent circuit used in EIS analysis and parameters extracted from fitting data in panel C.

Cyclic voltammetry (CV) and electrochemical impedance spectroscopy (EIS) were used for electrochemical characterization of the electrode after the polymer brush grafting and enzyme immobilization. We observed the presence of clear oxidation (right) and reduction (left) peaks in CV sweeps, both after grafting of the polymer brush and after immobilization of GOx (Figure 3A). All CV data was measured with 1 mM ferrocenemethanol as a redox active probe in ordinary (1×) PBS buffer. The peak-to-peak separation of the oxidation and reduction events did not change significantly: 77.0 mV for the bare gold, 75.4 mV for the surface modified with the polymer brush, and 79.1 mV after enzyme immobilization, using a scan rate of 25 mV/s (see additional CV data in Figure S1). This shows that the surface modification does not influence the redox probe accessibility and the values are close to the theoretical ideal peak separation of one-electron transfer reactions (59.2 mV).^39^ The reason why the enzymatic brush is not clearly detected is simply that even after the brushes are loaded with enzymes they remain highly solvated, as shown by the SPR results above (Figure 2C). Furthermore, the thin grafting layer prepared from a diazonium salt (characterization in Figure S2) has a negligible effect on the charge transfer, while thiolated initiators significantly block charge transfer and show poorer stability (Figure S3).

For the unmodified brush, we noted a significant increase in current and a broadening of the oxidation region from −100 to +300 mV, as well as a broadening of the reduction region from +150 to −200 mV. This is likely due to interactions between the redox probe and the brush, as the neutral ferrocene turns into the positive ferrocenium ion upon oxidation,^40^ which can act as counterion to the carboxylate groups of the polymers and thus remains at the surface. Furthermore, the oxidation peak current was linear with the square root of the scan rate (Figure 3B), indicating that the process is diffusion controlled and that the Randles-Sevcik equation for the peak current is valid:^39^
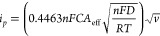
1Here *n* is the number of exchanged electrons per event (one for the ferrocene oxidation), *F* is Faraday’s constant, *A*_eff_ is the effective area of the working electrode, *C* is the bulk concentration, *D* is the diffusion coefficient, *R* is the ideal gas constant, and *v* is the scan rate. Using the geometric electrode area for *A*_eff_, the value of *D* calculated for the bare gold electrode is 6.71 × 10^–6^ cm^2^/s, which is comparable to what is reported in literature for ferrocenemethanol (7.8 × 10^–6^ cm^2^/s).^40^ After GOx is immobilized, *D* is similar to the value obtained for bare gold, which strongly suggests that the observed increase in current for the unmodified brush is due to its negative charges as the film should be closer to neutral after conjugation with GOx.

EIS was used to further investigate the electrochemical availability of the electrode after the surface modifications. Figure 3C shows Nyqvist plots for bare gold, after synthesizing the polymer brush and after immobilizing GOx. (The corresponding Bode plots are shown in Figure S4.) The equivalent circuit shown in Figure 3D was used to model the spectrum. It consists of an electrolyte resistance (*R*_s_) in series with a parallel combination of a capacitor and resistor that corresponds to the polymer coating,^22^ followed by the double-layer capacitance (*C*_dl_) in parallel with the charge transfer resistance (*R*_ct_) and a mass transfer (Warburg) impedance element (*Z*_w_).^41^ (For the bare gold, the components that correspond to the brush were not included.) After surface functionalization, there was no significant difference in *Z*_W_, which shows that the diffusivity in solution remains unaltered, as expected. An extremely small increase in *R*_ct_ (approximately from 1 to 2 Ω) was obtained after forming the polymer brush, and following the immobilization of GOx (∼1 Ω more). Also, the resistance of the brush itself is <1 Ω. These values confirm the surface functionalization qualitatively, but clearly the enzymatic brushes do not significantly reduce the electrode accessibility for redox reactions. This is partly because of the high degree of hydration of the layer, but also because the grafting layer with aryl bonds does not limit charge transfer significantly. As a comparison, when using other initiators based on conventional self-assembled alkanethiols, *R*_ct_ increments in the range 10^3^–10^5^ Ω have been reported by Anthi et al. for zwitterionic brushes,^20^ even without any enzymes present. Similarly, Panzarasa et al. reported resistance increases in the kiloohm range for poly(methacrylic acid) brushes.^42^ These values are many orders of magnitude higher than ours. In fact, the characteristic semicircle in the Nyqvist plot^22^ is not even visible in our system. Based on these results we refer to the enzymatic brushes as close to “electrochemically invisible”, i.e. they can barely be detected and the attenuation of redox activity is negligible. The extra capacitance from the brush (Figure 3D) is attributed to its charges and polarizable groups.^22^ In addition to the detailed characterization by EIS, we also tested the stability of the enzymatic brushes after 60 CV sweeps. The peak separation showed no change and the SPR spectra showed only a minor loss of mass from the surface (Figure S5).

### Electrochemical Glucose Detection

To evaluate the polymer brush interface for electrochemical sensing, we utilized amperometric detection of H_2_O_2_ by reductive potentials^43^ according to
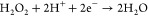


**Figure 4:**
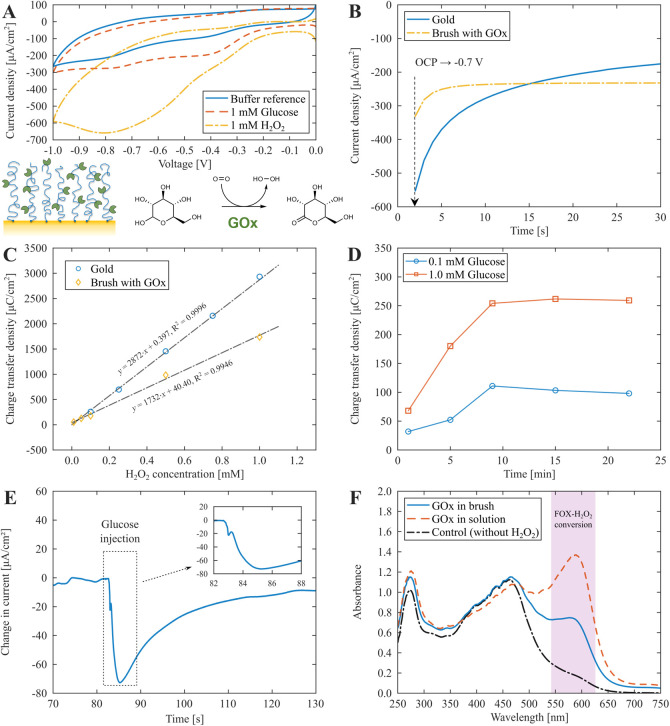
Electrochemical detection of glucose. (A) Current response from cathodic sweeps (100 mV/s) in PBS buffer, with 1 mM H_2_O_2_ or glucose added. (B) Representative chronoamperometry trace from detection of H_2_O_2_ present at 1 mM in bulk solution. The background current in the absence of H_2_O_2_ has been subtracted. (C) Integrated current from 25 s chronoamperometry recordings with different concentrations of H_2_O_2_ added to a bare electrode and an electrode modified with an enzymatic brush. The linear regressions are shown with *R*^2^ values. (D) Representative integrated chronoamperometry (25 s) signals vs time for two different concentrations of glucose added to a brush with GOx. (E) Real-time amperometry response to the injection of 1 mM glucose solution onto the enzymatic brush electrode. (F) Independent colorimetric verification of H_2_O_2_ production by immobilized GOx at the sensor surface compared to the response by the same quantity of GOx when free in bulk solution. The absorbance increase at 580 nm is due to generated H_2_O_2_.

GOx was first used as a model enzyme for testing the feasibility of enzymatic conversion and electrochemical sensing. The current response from negative voltage sweeps in glucose solutions and to reference solutions of H_2_O_2_ is shown in Figure 4A. The electrode gave a clear response from both 1 mM glucose and 1 mM H_2_O_2_ in comparison with the PBS buffer, for which the current is dominated by O_2_ reduction.^27^ Reduction of H_2_O_2_ reached a maximum contrast, relative to the background of PBS, at −0.7 V (vs Ag/AgCl).

For our detection concept, it is important to note that even with instant enzymatic conversion with 1:1 stoichiometry for glucose to H_2_O_2_, the current response from directly adding H_2_O_2_ to the solution is still expected to be higher than that from the same concentration of glucose (or any other analyte). This is because the addition of H_2_O_2_ is done by exchanging the entire bulk solution with a known concentration, while for the case of glucose, H_2_O_2_ is only generated at the interface. Hence, when the potential is applied, the concentration profile of H_2_O_2_ will not be uniform and less amounts are available compared to when the bulk concentration is altered. We refer to the Supporting Information including Figure S6 and eqs S1–S3 for an extended discussion on this point.

Using chronoamperometry at −0.7 V (example in Figure 4B), we observed a linear relationship between H_2_O_2_ concentration and the total charge transfer, for both the bare gold and the brush with GOx (Figure 4C). This is expected from the integrated Cottrell equation for purely diffusion-controlled Faradaic reactions on a planar electrode:^27^
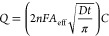
2Here *n* = 2 for H_2_O_2_ reduction and *Q* is the integrated current. The linear slope was observed to decrease from 2.87 CM^–1^ cm^–2^ for clean gold to 1.73 CM^–1^ cm^–2^ for the brush with GOx. This may be partly attributed to a small reduction in diffusivity and electrode accessibility due to the enzymatic brush coating, in qualitative agreement with the EIS results (Figure 3D). However, we mainly attribute this to the well-known inhibitory interactions between H_2_O_2_ and GOx.^44^ If the molecule binds to the enzymes it cannot easily react with the gold surface underneath. This explains why the reduction of redox activity appears much stronger than for ferrocenemethanol (Figure 3). Indeed, the kinetics of the current trace did not fit the Cottrell expression in this case, while performing the same experiment on brushes with other enzymes showed kinetics that followed the Cottrell expression perfectly, just as for an unmodified electrode (analysis in Figure S7).

The electrodes with GOx brushes were further exposed to glucose solutions of varying concentrations and repeated chronoamperometry measurements were performed over time (Figure 4D). The response, measured as the integrated current, increased over time, showing that the local H_2_O_2_ concentration increases when the enzymes are given more time to operate. Notably, the sensor signal can thus be enhanced simply by delaying the readout. It is also noteworthy that the signal increases even though all H_2_O_2_ close to the surface is consumed for each data point in time in Figure 4D. As expected, increasing the glucose concentration from 0.1 to 1.0 mM also resulted in a higher signal, although not by a factor of 10. This is again attributed to the relatively strong inhibition of GOx by the generated H_2_O_2_ expected in this concentration range.^44^

For certain applications (e.g., on living cells), the sensor will need to respond fast to detect transient increments in analyte concentration.^12^ To get an estimate of the response time of the enzymatic brush electrodes, chronoamperometry was performed with a 20 ms time resolution (Figure 4E). Upon injection of glucose onto the electrode, the response appeared instantly (<1 s), reaching its maximum value after 2.4 s. This demonstrates that time-resolved measurements are feasible with the electrode interface, although further studies using higher bandwidth potentiostats are needed to precisely determine the response kinetics. Additionally, the enzyme loading and the polymer brush thickness are likely important parameters to alter when optimizing sensor speed,^21^ but this is beyond the scope of the present study.

We independently verified the enzymatic reaction using a colorimetric assay^45^ for H_2_O_2_ (Figure 4F), where the absorbance is proportional to the concentration of H_2_O_2_ in the sampled solution (details in Figure S8). Since the amount of GOx on the surface was determined from SPR (Figure 2), we could compare its activity with the same amount of GOx in solution phase. The results indicated that the specific activity of GOx conjugated to PAA brushes was 43% lower than in solution. While enzymes generally suffer activity loss upon immobilization,^16^ GOx stands out as being particularly robust^34^ and can maintain full activity.^46^ We also tested the specific activity of GOx directly adsorbed on gold and found that it was slightly higher (51% of bulk, Figure S8), which contradicts our previous study.^18^ We believe the main explanation for the apparent lower activity observed for the brushes is that they are so heavily loaded with GOx that substrate depletion may become significant (see Figure S9 and related discussion). Indeed, similar reductions in specific activity have been reported for other surfaces when they become heavily loaded with GOx.^47,48^ Regardless, even if the enzymes actually only maintain ∼50% of their activity in bulk, this is more than sufficient to create an efficient catalytic interface due to the high mass coverage.

### Neurotransmitter Detection

**Figure 5:**
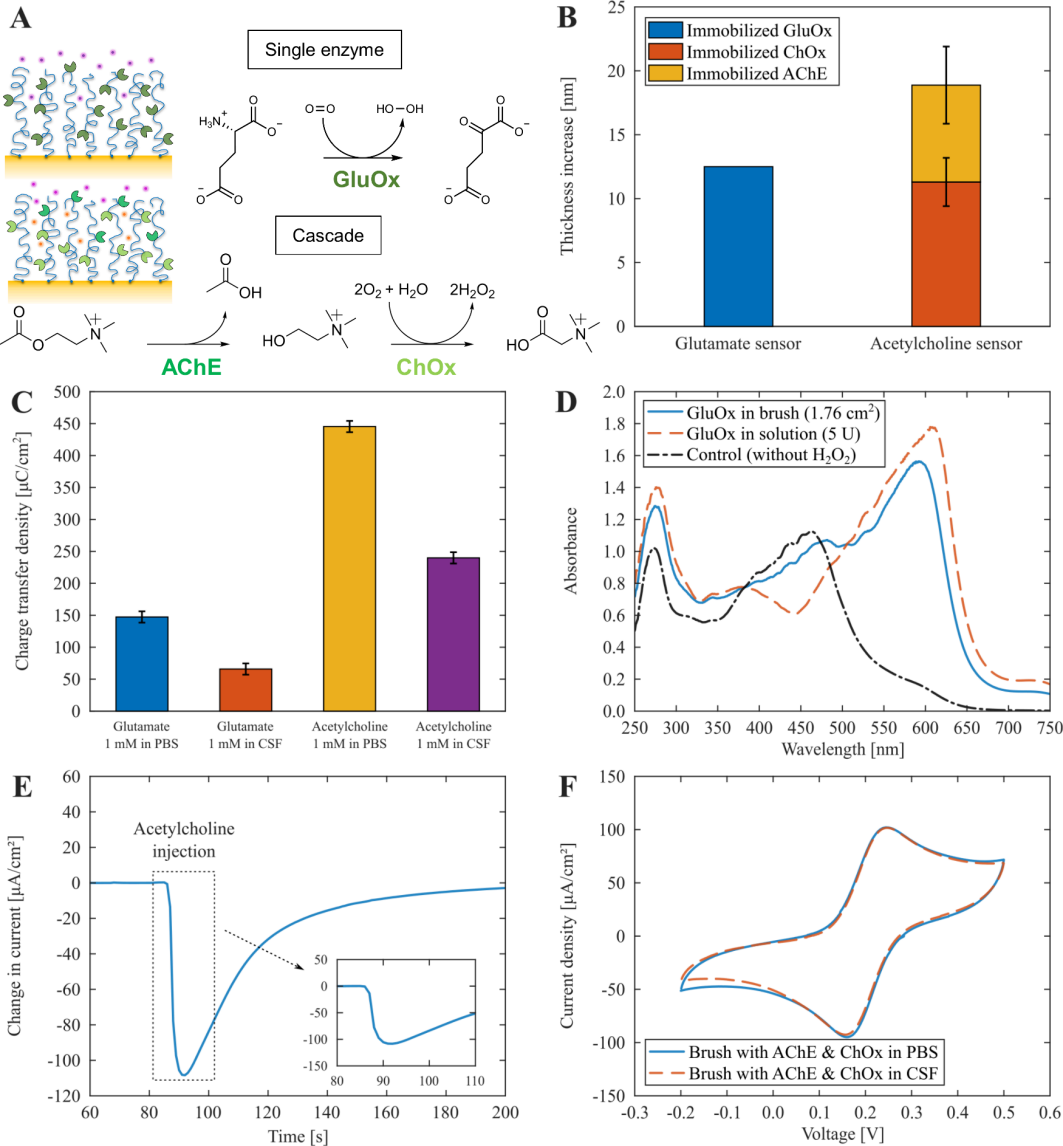
Neurotransmitter detection in biofluids. (A) Reaction schemes for H_2_O_2_ generation using either one (glutamate) or two (acetylcholine) enzymes. (B) Immobilized amount (dry film thickness) of GluOx and ChOx + AChE after polymer brush functionalization. The PAA dry thickness was ∼20 nm in all cases. (C) Integrated currents from chronoamperometry at −0.7 V (for 25 s) for detection of glutamate and acetylcholine using brushes with GluOx or ChOx + AChE (5:1 molar ratio) respectively. The background signals measured before analyte addition have been subtracted. Error bars represent the instrumental variation from repeated measurements. (D) Colorimetric verification of GluOx activity in the polymer brush and in solution phase. (E) Real-time detection of acetylcholine (1 mM injected over the surface) using the cascade reaction system. (F) CV data for a brush functionalized with AChE and ChOx in PBS and in contact with CSF (1 mM ferrocenemethanol, scan rate 25 mV/s).

To demonstrate the versatility of the enzymatic polymer brush interface we tested detection of two neurotransmitters, glutamate and acetylcholine, which are important biomarkers for neurodegenerative diseases as well as other conditions (e.g., obesity for glutamate and hypertension for acetylcholine). In both cases, the enzymes involved generate H_2_O_2_ by catalyzing oxidative reactions. For glutamate detection, glutamate oxidase (GluOx) converts l-glutamate to α-ketoglutarate and H_2_O_2_.^4,11^ For acetylcholine, a cascade system consisting of acetylcholine esterase (AChE) and choline oxidase (ChOx)^10,36,37^ converts acetylcholine to glycine betaine and 2H_2_O_2_ (Figure 5A). Quantification of immobilized enzymes, electrochemical surface characterization and amperometric responses were measured in the same manner as for glucose detection with GOx. As expected, immobilization was successful for all enzymes (Figure 5B) with an average immobilized mass of approximately 1.4–1.5 μg/cm^2^ (Table S1). Starting with glutamate, the integrated current from chronoamperometry at −0.7 V gave a clear response from 1 mM analyte compared to values obtained in PBS (Figure 5C). As with glucose, the sensor response (and thus the detection limit) depended on how long time the analyte was allowed to react (Figure S10). No signals were observed in the absence of enzymes as expected since the analytes are not electro-active.

Furthermore, we again measured the activity of the enzymes conjugated to the PAA brush using the colorimetric assay for H_2_O_2_. For GluOx, we quantified the amount of H_2_O_2_ produced by enzymes with known activity in solution (5 U) and compared with a sensor surface containing 3 μg GluOx (Figure 5D). The surface converted ∼15% of the available glutamate in a 2 mL vial in 10 min. Based on this result, the specific activity of the immobilized GluOx was calculated to 2.5 U/mg (4.1 mU/cm^2^), which closely matches literature values for GluOx in solution phase.^49^ The value is also higher than what we obtained for immobilized GOx (1.6 U/mg based on data in Figure 4F). For acetylcholine, a direct comparison with enzymes in solution is not straightforward since proximity effects influence the total conversion rate under non-steady-state conditions,^50^ so the assay was only used to verify the reaction qualitatively (not shown). Nevertheless, these results demonstrate that the soft polymer brush is an excellent immobilization scaffold not only because it allows multilayers of enzymes to be assembled, but also because they retain high activity. This is likely is due to good substrate access and high conformational freedom inside the hydrophilic brushes.^16,47^ For comparison, the direct adsorption of AChE onto an electrode has been reported to reduce specific activity by nearly a factor of 10.^37^

Detection of acetylcholine requires two enzymes (AChE and GluOx) providing an opportunity to evaluate the performance of the enzymatic polymer brushes for cascade reactions. To the best of our knowledge, cascade reactions have never previously been performed using polymer brushes as scaffolds, while several other constructs have been reported.^51^ In cascade reactions, the relative amounts of the enzymes are expected to strongly influence the total reaction rate.^50^ With our conjugation method, it is not obvious whether the enzymes should be introduced simultaneously or in sequence to the brush. For this initial study, we chose to introduce them sequentially since this allowed us to quantify the amount of each enzyme by SPR. Furthermore, as AChE has a considerably higher molecular weight (280 kg/mol) than ChOx (95 kg/mol), we expected a greater immobilization capacity of AChE due to more hydrogen bonds during the immobilization,^17^ prior to the formation of covalent amide bonds that lock the protein in place. Hence, we first immobilized ChOx for 10 min, followed by AChE. Indeed, as shown in Figure 5B, this approach led to large amounts of immobilized AChE (1.0 μg/cm^2^) even though the brush was already highly loaded with ChOx (1.5 μg/cm^2^). This corresponds to ∼ 5 times more ChOx than AChE in terms of molar ratio. It should be noted, however, that this ratio may not be optimal for maximizing the total reaction rate and the speed of the sensor. Previously, a ratio of 1:10 for AChE:ChOx was used for enzymes adsorbed directly on the solid electrode.^36^ For future optimization, the ratio can be easily adjusted by altering the immobilization time for the first enzyme.

The cascade reaction for acetylcholine detection produced an even higher amperometry signal in comparison with glutamate at the same analyte concentration (Figure 5C). This is partly because two H_2_O_2_ molecules are generated for each analyte instead of one, but the increase is more than a factor of 2, indicating that the cascade reaction runs very efficiently. We also tested the sensor response upon injection of acetylcholine over the electrode surface, which resulted in an immediate response in chronoamperometry (Figure 5E). The sharp increase in current magnitude revealed by the kinetics confirms a fast enzyme conversion also for the two-step cascade.

We also evaluated the enzymatic polymer brushes for sensing in complex media. Both the glutamate and acetylcholine sensors successfully detected their respective analytes in cerebrospinal fluid (CSF). Note, however, that the CSF had to be diluted 10 times simply to enable proper flow into the liquid cell. Also, the signals were reduced in comparison with detection in pure PBS (Figure 5C), suggesting some interference from the biofluid. For instance, reactions with antioxidants, such as ascorbic acid (vitamin C) which is naturally present in CSF,^52^ would remove a fraction of the H_2_O_2_ generated by the enzyme and thus lower the overall signals. This explains why the relative signal reduction is almost the same for both neurotransmitters (Figure 5C). To confirm that the electrode was still equally redox-active, CV was performed in 10× diluted CSF at pH 7.5 (Figure 5F). The voltammograms were almost identical before and after exposure to CSF, showing that the electrode reactivity was not significantly altered. EIS was measured as well and revealed only minor changes in the electrode characteristics (Figure S4). Hence, the electrode functions well in CSF, which can be attributed to the polymer brushes preventing large molecules from reaching the metal surface. However, PAA and the enzymes conjugated to it is not the most repelling brush and SPR data showed that some species from CSF did bind (Figure S11). Since there was no negative impact on redox reactions, these interactions must occur in the upper regions of the brush, i.e. the brush is still fully antifouling in the sense that the solid surface is not affected and measurements in complex biofluids are clearly possible. While other constructs have been reported for this purpose,^14,15^ they do not have conjugated enzymes. Thus, our enzymatic polymer brushes represent a new and generic modification strategy where enzymes can be immobilized in very high quantities and securely grafted through covalent bonds.

## Conclusions

In this work, surface sensitive and electrochemical characterization techniques were employed to investigate the viability of a novel electrode interface combining the antifouling characteristics of polymer brushes with enzyme-enhanced biomarker detection. Using generic conjugation chemistry, we covalently bound large amounts of enzymes to the polymer brush. In contrast to previous work on neurotransmitter sensors, we focused on a thorough quantitative characterization of the interface and its enzymes. We demonstrated the functionalization and conjugation of GOx, GluOx, ChOx, and AChE to the interface, for detection of glucose, glutamate and acetylcholine. Importantly, the electrode accessibility was not significantly altered by the presence of the polymer brush or its enzymes. We also demonstrated that the specific activity of the enzymes is not strongly reduced after incorporation into the polymer brush. Most importantly, detection works even in a biofluid, albeit with reduced signals. Further work will focus on various optimization aspects, for instance with respect to the amount of immobilized enzymes and the total thickness of the brush.

We emphasize that this study presents a versatile method for using polymer brushes as a matrix for immobilization of enzymes on electrodes, opening up for a wide range of applications in electrochemical sensing. While this work focused on neurotransmitter oxidative enzymes, the adaptable design allows for the detection of numerous nonelectroactive species, assuming an appropriate enzyme is available for enzymatic breakdown of the analyte into an electroactive product. This research advances the development of robust and sensitive biomarker detection techniques. As a next step, potentiostats/amplifiers designed for low-noise measurements should be implemented and the surface modification protocol should be adapted to microelectrodes for high-speed monitoring of neurotransmitter activity at synapses.^12^ As an alternative application direction, there is also potential for scaling up the enzymatic brushes for efficient biocatalytic synthesis of valuable compounds.

## Experimental Section

### Chemicals

All chemicals used were purchased from Sigma-Aldrich unless stated otherwise. Water used was ASTM research grade Type 1 ultrafiltered water (MQ, 18.2 MΩcm). Hydrogen peroxide (H_2_O_2_, 30%) and ammonium hydroxide (NH_4_OH, 28–30% in water) were from ACROS chemicals or Thermo-Fischer Scientific. Chemicals used for diazonium salt grafting were 4-aminophenetyl alcohol, tetrafluoroboric acid solution (HBF_4_), *tert*-butyl nitrite and ascorbic acid. Chemicals used for polymer synthesis were triethylamine, α-bromoisobutyryl bromide, CuCl_2_, *tert*-butyl acrylate, N,N,N′,N″,N″-pentamethyldiethylenetriamine, ascorbic acid and methanesulfonic acid. Chemicals used for the enzyme immobilizations were 2-(N-morpholino)ethanesulfonic acid hydrate (MES), 1-ethyl-3-(3-(dimethylamino)propyl)carbodiimide (EDC) and *N*-hydroxysuccinimide (NHS).

Electrochemical measurements were done with ferrocenemethanol (98%). Ferrous oxidation-xylenol orange (FOX) assays used were Pierce Quantitative Peroxide Assay Kits, from Sigma-Aldrich. Enzyme activities were recorded with d-glucose, acetylcholine chloride, and l-glutamic acid monosodium salt hydrate. Unless stated otherwise, the buffer used in all measurements was (1×) phosphate buffered saline (PBS; 10 mM phosphate, 137 mM NaCl and 2.7 mM KCl) at pH 7.5. The pH was adjusted with 1 M HCl or 1 M NaOH solutions and was controlled within ± 0.05.

The enzymes used were: GOx Type VII from *Aspergillus niger* (Sigma-Aldrich product G2133), with a molecular weight of 160 kDa and an isoelectric point of 4.2. ChOx from *Alcanligenes* sp., (Sigma-Aldrich product C5896), with a molecular weight of 160 kDa and an isoelectric point of 4.1 ± 0.1. AChE Type VI–S from *Electrophorus electricus* (Sigma-Aldrich product C3389), with a molecular weight of 160 kDa and an isoelectric point of 5.5. GluOx from *Streptomyces* sp. (Sigma-Aldrich product G5921), with a molecular weight of 120 kDa and an isoelectric point of 8.5. Molecular weights and isoelectric points are given as stated by the supplier, except for GluOx whose values were previously stated by Wachiratiancha et al.^53^

### Diazonium Salt Synthesis

4-(2-Hydroxyethyl)phenyldiazonium tetrafluoroborate was synthesized using a modified version of our previously reported method.^27^ 4-aminophenethyl alcohol (0.22 g, 1.60 mmol) was dissolved in HBF_4_ (0.3 mL, 48%) and was diluted with MQ water (1 mL). The solution was cooled in an ice bath and *tert*-butyl nitrite (0.2 mL, 1.70 mmol) was added dropwise while stirring. The solution was left to stir for 1 h. Using this method, a complete conversion of the amine was achieved (Figure S2). Hints of degradation product was observed in the ^1^H NMR, however, the final polymer brushes appeared identical compared to the previous method.^27^

### Surface Preparation

All electrodes and SPR sensor chips were manufactured in the same manner by depositing metal on glass substrates. Some SPR and electrochemistry experiments were also performed on the very same surface (e.g., Figures S3 and S5). Surfaces were cleaned with RCA1 wash consisting of a 1:1:5 volume ratio NH_4_OH, H_2_O_2_ and water for 20 min at 75 °C, rinsed with MQ water and ethanol, and dried under flow of N_2_ and then cleaned with UV O_3_ (placed under a 90 W mercury vapor lamp for 10 min) prior to immobilization of the initiation layer.

### Surface Activation

Ascorbic acid (0.035 g, 0.20 mmol) was dissolved in water (50 mL) and the solution was deoxygenated with N_2_ for 1 h. The solution was the transferred into a sealed glass jar with cleaned surfaces. The diazonium salt was deoxygenated with N_2_ for 5 min and transferred to the jar via needle. Surfaces were exposed to the diazonium salt for 1 h and then rinsed with MQ water and ethanol and dried under flow of N_2_. To convert the diazonium salt into 4-(phenethyl 2-bromo-2-methylpropanoate), the surfaces were exposed to α-bromoisobutyryl bromide (0.500 mL, 4.05 mmol) and triethylamine (0.675 mL, 4.84 mmol) in dichloromethane (50 mL) for 15 min, followed by rinsing in ethanol and drying under flow of N_2_. X-ray photoelectron spectroscopy, performed as described previously,^54^ was used to characterize the ATRP initiator layer (Figure S12).

### Surface-Initiated Polymerization

Surfaces for electrochemistry and SPR were polymerized in parallel to ensure the same kind of organic films were analyzed with both methods. Atom transfer radical polymerization (ATRP) with activators regenerated by electron transfer (ARGET) was used to synthesize PAA brushes on the 4-(phenethyl 2-bromo-2-methylpropanoate) initiation layer. CuCl_2_ (0.0053 g, 0.04 mmol), and PMDETA (0.065 mL, 0.31 mmol) were dissolved in dimethyl sulfoxide (20 mL). Toluene (12 mL) and *tert*-butyl acrylate (10 mL, 68.00 mmol) were added, and the solution was deoxygenated with N_2_ for 1 h. The reaction solution was then transferred via cannula into a screw-top jar (with rubber septa lid) containing initiator-prepared gold surfaces. The reaction was initiated by the addition of ascorbic acid (0.045 g, 0.26 mmol) and was quenched after 30 min by immersing the surfaces in ethanol. To convert the poly(*tert*-butyl acrylate) brushes into PAA brushes the surfaces were exposed to methanesulfonic acid (0.300 mL, 4.62 mmol) in dichloromethane (50 mL) for 15 min, after which surfaces were rinsed in ethanol and dried under flow of N_2_. Chemical characterization was performed by FTIR spectroscopy (Figure S13).

### Enzyme Immobilization

Covalent immobilization of GOx, GluOx, ChOx and AChE to PAA brushes was performed with EDC/NHS cross coupling using EDC (5 mM) and NHS (10 mM) in 10 mM MES (pH 5.0) for 45 min. GOx samples were prepared by immersing the surfaces in 0.5 g/L GOx in MES buffer for 1 h. Cascade samples were prepared by first immersing the surfaces in 0.2 g/L ChOx in MES for 10 min, rinsing with MES buffer, and immersing in 0.2 g/L AChE for 1 h. After exposure to enzymes all surfaces were rinsed with MES buffer and immersed in PBS buffer (150 mM, pH 7.5) for 20 min. Surfaces were stored at 8 °C in PBS buffer until use, but never for longer than a day after immobilization. To quantify the AChE and ChOx amounts separately, SPR measurements were performed on surfaces that had both enzymes and on surfaces where only the ChOx step had been done.

### Surface Plasmon Resonance

Measurements were performed on a SPR Navi 220A instrument (BioNavis), both in air and in water. The total internal reflection (TIR) and SPR angle were monitored with the 785 and 980 nm laser diodes, respectively. The flow rate of buffer used was 10 μL/min, unless otherwise stated, and all measurements were done at 25 °C. The methodology of analyzing SPR spectra by Fresnel modeling and the quantification in dry state has been described in previous work.^55^ The noninteracting probe method was used to determine the exclusion height^55^ (hydrated thickness) of the PAA brush before and after protein immobilization, with 20 mg/mL 35 kDa PEG as probe. The refractive index used for PAA was 1.527 and the refractive index of all enzymes was assumed to be equal to this value. Calculation of the surface coverage was done with the density of the bulk polymer material (1.41 g/cm^3^) and an average value for proteins (1.35 g/cm^3^). While SPR was used to obtain values of dry thickness for all layers, we also verified that ellipsometry gave consistent results (Figure S14) using a J.A. Woollam RC2 spectroscopic ellipsometer.

### Electrochemical Characterization

CV and EIS measurements were performed with a Reference 600+ (Gamry Instruments) potentiostat. A conventional three-electrode setup was used, with the gold substrate as the working electrode, a platinum wire mesh as counter electrode, and a Ag/AgCl electrode as reference. Measurements were performed with 1 mM ferrocenemethanol in a 150 mM PBS solution (pH 7.5). CV was used to determine the standard redox potential (*E*^0^) of the redox probe as the average of potential values at which the cathodic and anodic currents were maximal. All CV sweeps were initiated with an anodic sweep from −0.2 V to +0.5 V, followed by a cathodic sweep from +0.5 V to −0.2 V, at 10, 25, 40, 60, 90, 160, 250 and 360 mV/s. Five cycles were run and the average of the last three were used to determine the cathodic and anodic current maxima as well as for the diffusional analysis. EIS was measured with a DC potential set at the *E*^0^ of ferrocenemethanol over frequencies ranging from 10^4^ to 10^–1^ Hz, with an AC amplitude of 5 mV. Three repeats were run, and the average of these was used for equivalent circuit fitting.

### Detection of H_2_O_2_ by Amperometry

Amperometry measurements were performed with the same electrochemical setup. Chronoamperometry was measured at −0.7 V. CV was run on each electrode before use to ensure solvent availability. All analytes were introduced in PBS at pH 7.5. The CSF measurements were done under the same conditions but with CSF 10× diluted in PBS. Charge transfer densities were determined by measuring amperometry and integrating during 25 s, ignoring the first 5 s when the potential is established. The sensors were exposed to their respective analyte solutions for 5 min unless otherwise stated.

### Optical Verification of Enzyme Activity

A FOX assay was used for optical quantification of H_2_O_2_ production by the enzymes.^45^ The FOX assay was prepared by mixing 0.5 mL of 25 mM ammonium ferrous (II) sulfate composition in 2.5 M H_2_SO_4_ with 50 mL of 100 mM sorbitol and 125 μM xylenol orange (*o*-cresol-sulfonephthalein-3′-3′-bis-[methyliminodiacetic acid sodium salt])) in water, and 2 mL of mixture was added to a vial containing 0.2 mL sample with H_2_O_2_. The sample was incubated for 20 min at room temperature and measured in a custom setup with a fiber coupled array spectrometer and lamp (BH-2000-BAL Deuterium-Halogen Light Source, Ocean Optics) with collimating lenses. Enzyme activities were measured with a 0.5 mM bulk solution of the substrate in PBS at pH 7.5. The same amount of enzyme, in total mass, of GOx was used on the surface and in solution. A solution of GluOx with total activity of 5 U according to supplier was used for measuring the activity in bulk.

## Supporting Information Available

Surface coverages for enzymes, additional CV data, NMR data, data on thiol initiator, additional EIS data, stability test, theory of concentration profiles, additional chronoamperometry data, description of colorimetric assay, substrate depletion effect, time dependence of signal from neurotransmitters, SPR data of fouling, XPS data, FTIR data and ellipsometry data (PDF)
