# Supplementary material for: Enzymatic Polymer Brush Interfaces for Electrochemical Sensing in Biofluids
Source: ACS Appl Bio Mater. 2025 Apr 24;8(5):4008–19. doi: 10.1021/acsabm.5c00146 (PMC12093368; doi:10.1021/acsabm.5c00146)
Supplement: Supplementary file 1 — mt5c00146_si_001.pdf [file mt5c00146_si_001.pdf]

Enzymatic Polymer Brush Interfaces for Electrochemical Sensing in Biofluids

*Jesper Medin,[1] Maria Kyriakidou,[2] Bagus Santoso,[2] Pankaj Gupta,[1] Julia Järlebark,[1] Andreas Schaefer,[1] Gustav Ferrand-Drake del Castillo,[2] Ann-Sofie Cans[1] and Andreas Dahlin.[1]**

1 Department of Chemistry and Chemical Engineering, Chalmers University of Technology, 41296 Gothenburg, Sweden.

2 Nyctea Technologies AB, AstraZeneca BioVentureHub, 431 83 Mölndal, Sweden.

* Corresponding author: adahlin@chalmers.se

Table S1 Quantification of enzyme immobilization (in µg/cm²) with SPR measurements in air, and the calculated amount of immobilized enzymes (enzymes/µm²). Surface coverages were calculated using known average molecular masses of the enzymes, with GOx at 160 kDa (n = 4), GluOx at 120 kDa (n = 1), ChOx at 95 kDa (n = 4), and AChE at 280 kDa (n = 4). No repeat measurements with SPR were done for GluOx because of limited enzyme availability. Note that the PAA grafting density cannot be determined since the molecular weight is unknown. (This is normally the case when "grafting from".)

Layer	Immobilized mass (µg/cm²)	Surface coverage (molecules/µm²)
PAA	4.319 ± 0.241	Unknown
GOx	2.690 ± 0.218	101 200 ± 8 200
GluOx	1.687	84 700
ChOx	1.526 ± 0.256	96 700 ± 16 200
AChE	1.023 ± 0.409	22 000 ± 8 800

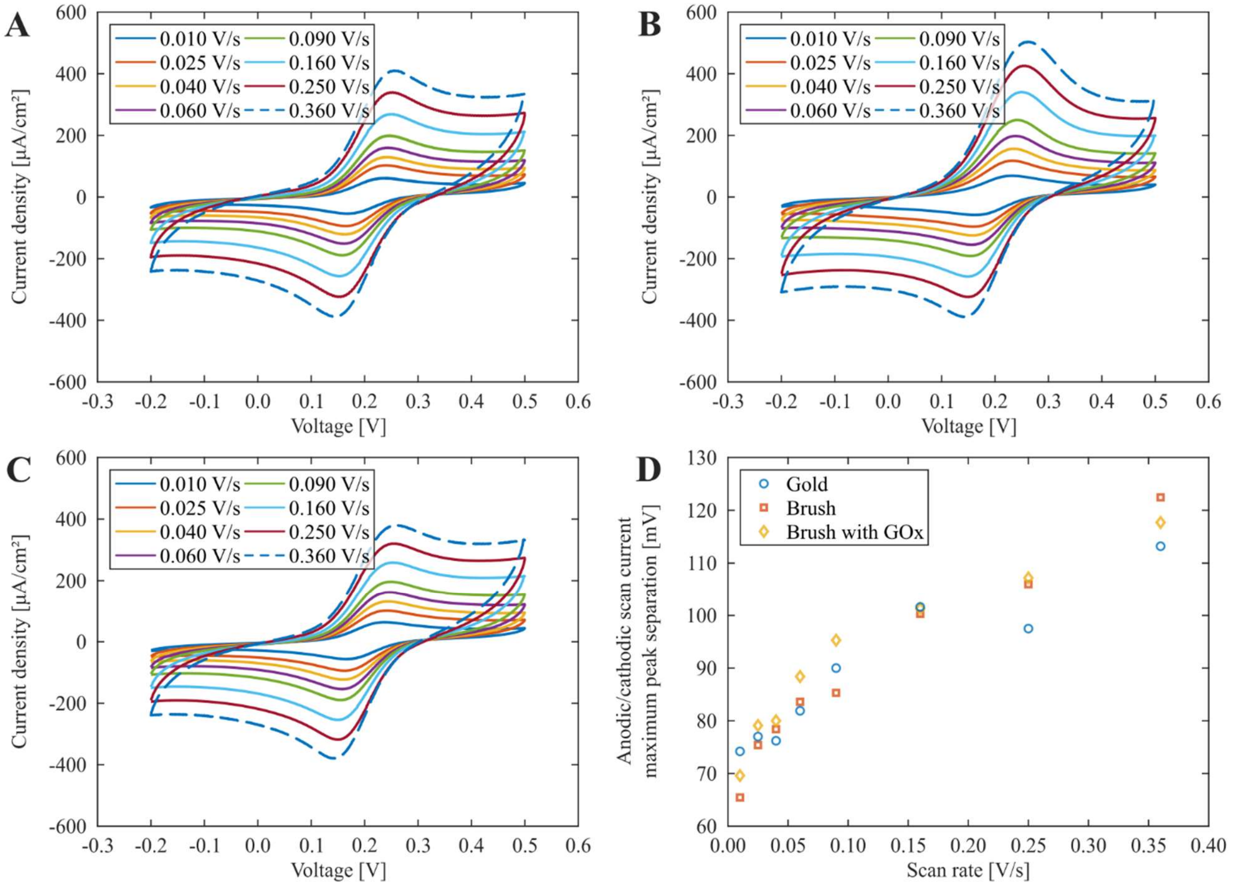

Figure S1 Additional CV data at different scan rates from 0.010 V/s to 0.360 V/s. (A) Bare gold. (B) After polymer brush formation. (C) After *ex situ* GOx immobilization. Higher current maxima are observed in the anodic sweep (from −0.2 V to +0.5 V) after the polymer brush formation for each scan rate compared to the bare gold or after GOx immobilization, while the current maxima in the cathodic sweep (from −0.2 V to +0.5 V) remain the same. (D) Anodic and cathodic current maximum peak separation for bare gold, after polymer brush formation and after GOx immobilization. A similar increase in peak separation was observed for each sample type for each scan rate, indicating similar electron transfer properties after polymer formation and enzyme immobilization compared to the bare gold substrate, for the ferrocenemethanol redox probe.

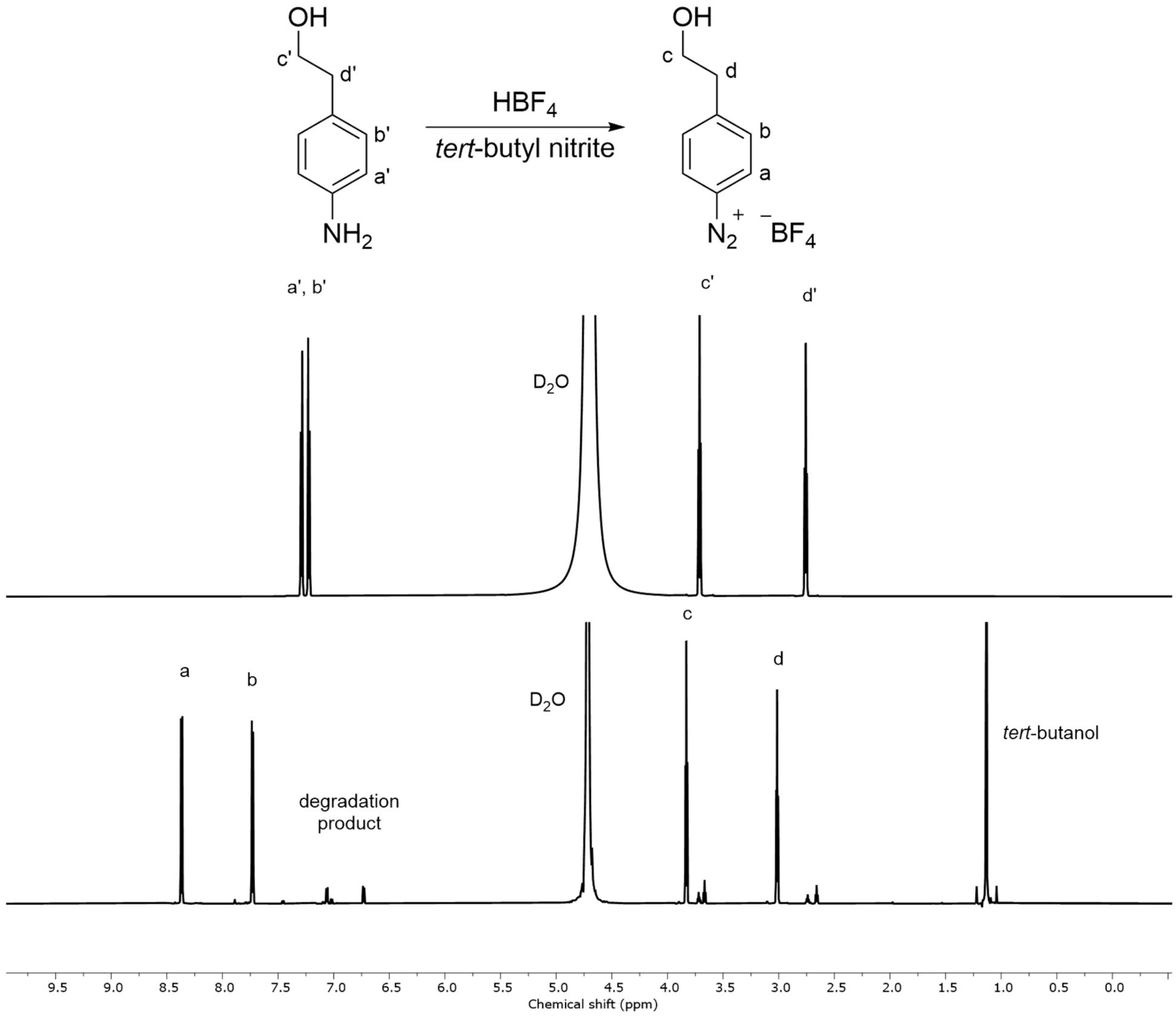

Figure S2 ^{1}H NMR spectra of 4-aminophenethyl alcohol (upper spectra) and 4-(2-hydroxy-ethyl)phenyldiazonium tetrafluoroborate (lower spectra) with peak assignment highlighted with the respective chemical structure. After the reaction the two peaks associated with the product was observed at 7.75 ppm and 8.35 ppm, as well as minor shifts in the two peaks associated with the ethanol group. The complete loss of the two peaks at 7.25 ppm in the lower spectra indicates close to full conversion of 4-aminophenethyl group into 4-(2-hydroxyethyl)phenyldiazonium.

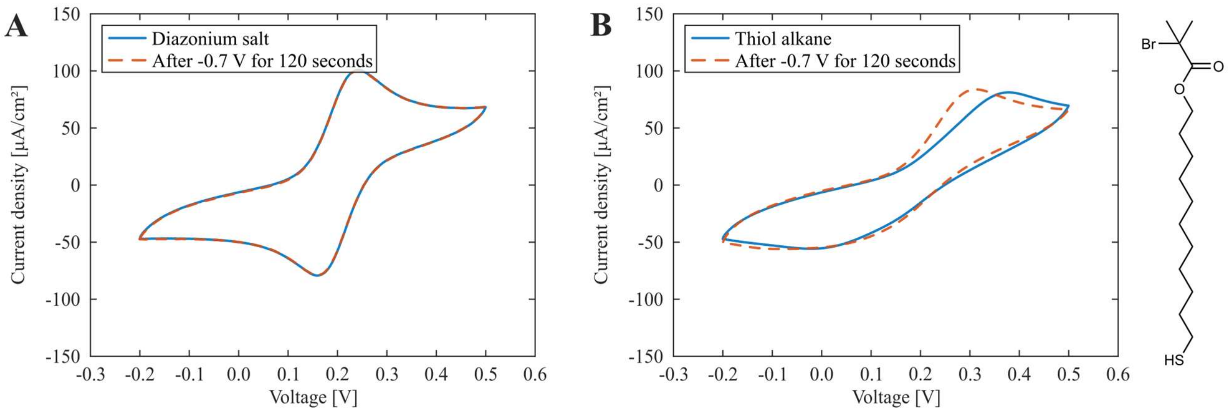

Figure S3 CV data on (A) the initiator layer and (B) a typical thiol-based initiator (structure shown). In the latter case, the charge transfer is significantly blocked. Also, after applying a reductive potential the curve changes due to desorption of the thiols, while the diazonium layer is stable. The thiol layer thickness on this sample was 1.7 nm.

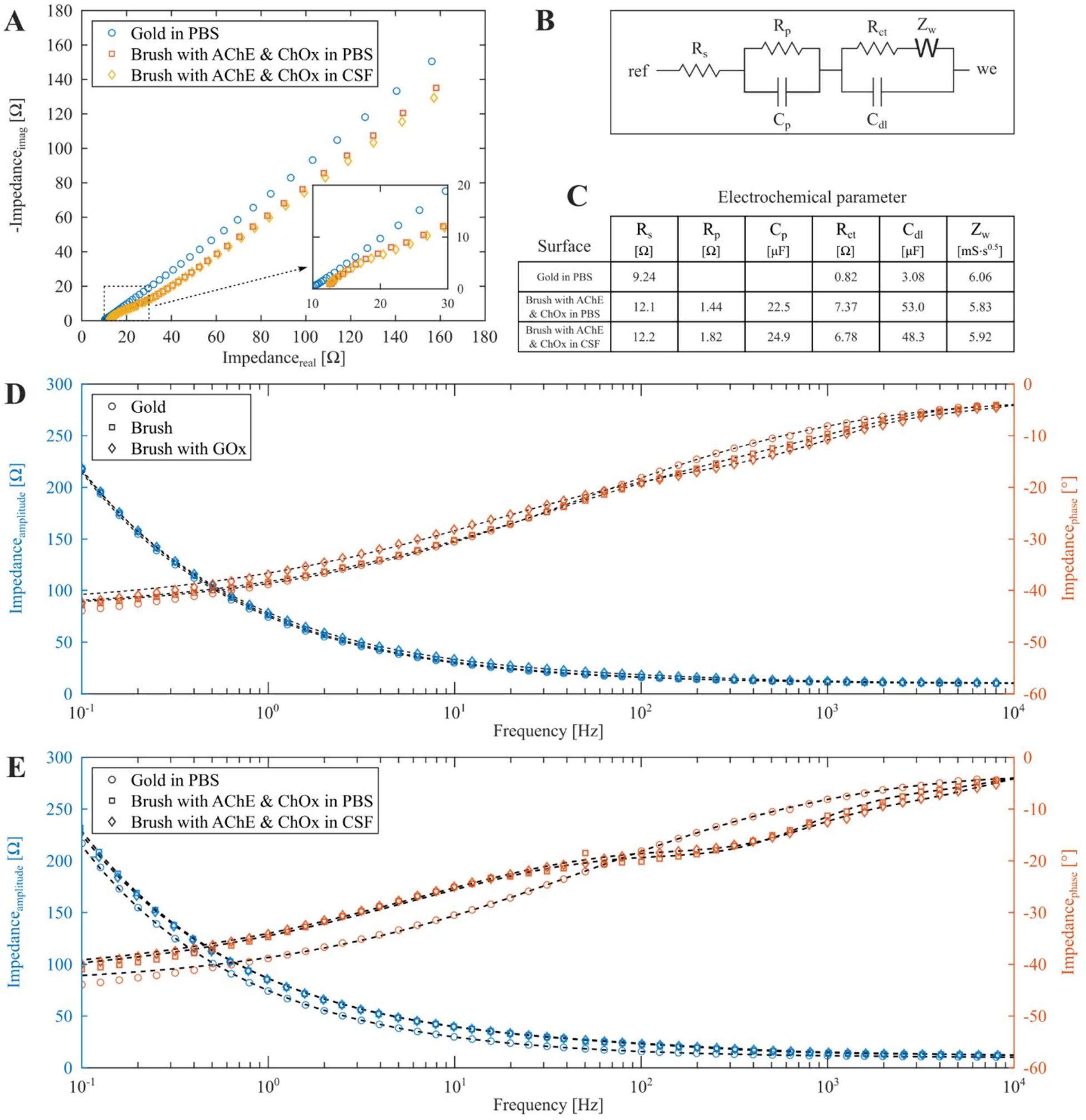

Surface	R_s [Ω]	R_p [Ω]	C_p [μF]	R_{ct} [Ω]	C_{dl} [μF]	Z_w [mS·s$^{0.5}$]
Gold in PBS	9.24			0.82	3.08	6.06
Brush with AChE & ChOx in PBS	12.1	1.44	22.5	7.37	53.0	5.83
Brush with AChE & ChOx in CSF	12.2	1.82	24.9	6.78	48.3	5.92

Figure S4 Additional EIS data. (A) Nyquist plots for enzymatic brush in PBS and in CSF. Each spectrum is the average of three repeats. (B) Equivalent circuit used in the analysis (see also Figure 3 in the main text). (C) Parameters extracted from fitting data to the equivalent circuit. (D) Bode plots for bare gold, after polymer formation and after immobilization of GOx. The data is equivalent to the Nyquist plots in the main text. Model fits are included. (E) Bode plots equivalent to the data in panel A and model fitting.

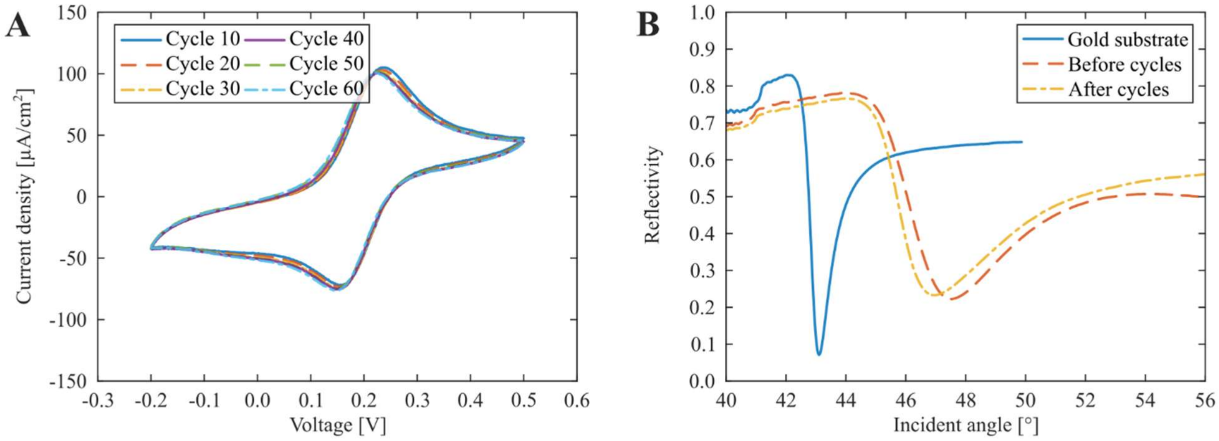

Figure S5 Stability test. (A) Repeated CV sweeps using a brush with GOx. (B) SPR spectra (of the same surface) recorded in air before and after the CV sweeps. The small loss in mass may be due to a small fraction of polymer chains not covalently grafted.

Theory of redox species concentration evolution in space and time

To estimate the concentration of the redox active species generated at the electrode by the enzymes, we first assume that the brush is thin (~100 nm) compared to the characteristic diffusion length over the timescale of the experiment (>100 μm) and that the brush does not represent a significant diffusion barrier, as shown by the results. This means that the products of the enzymatic reaction are generated just at the surface, and we can use expressions derived for the current in voltammetry under diffusion-limited conditions.[1] The substrate and products in enzymatic reactions are typically small and we can assume they have similar diffusion coefficients, in which case analytical solutions emerge.[2] The (not redox active) analyte concentration will be given by:

$$C(z,t) = C_0\left[1 - \mathrm{erfc}\left(\frac{z}{2\sqrt{Dt}}\right)\right] \qquad \text{(S1)}$$

Here C_0 is the bulk concentration in the sample and erfc is the complementary error function:

$$\mathrm{erfc}(x) = 1 - \frac{2}{\sqrt{\pi}}\int_0^x \exp(-\tau^2)\mathrm{d}\tau \qquad \text{(S2)}$$

The concentration of the redox active species (in our case H_2O_2) will be given by:

$$C_{\mathrm{redox}}(z,t) = C_0 - C(z,t) = C_0 \times \mathrm{erfc}\left(\frac{z}{2\sqrt{Dt}}\right) \qquad \text{(S3)}$$

Note, that this is the solution to a mass transport problem and the enzymatic conversion to the redox active species is assumed to be instant once the substate reaches the surface with the polymer brush. (The result actually has nothing to do with electrochemistry in principle: it is the enzymes that convert the molecules and not the electrode.) An example of the concentration profile of the analyte and the redox active molecule (1:1 conversion) is shown in Figure S6 for $D = 10^{-9}$ m^2/s and $z < 1$ mm after $t = 1$ min and $t = 10$ min.

Here it is important to note that the concentration of H_2O_2 at the electrode will be equal to that of the analyte in the bulk, i.e. $C_{\mathrm{redox}}(z = 0, t) = \mathrm{C}(z \rightarrow \infty, t) = C_0$. However, the total amount of H_2O_2 available to react with the electrode in chronoamperometry is not the same as when performing a calibration, where certain concentrations of H_2O_2 are introduced in the bulk. The latter gives a constant concentration throughout the solution when the potential is applied. Given

---

[1] Bard, A. J.; Faulkner, L. R., *Electrochemical methods: fundamentals and applications*. 2nd ed.; Wiley: New York, **2001**.

[2] Compton, R. G.; Laborda, E.; Ward, K. R., *Understanding voltammetry: simulation of electrode processes*. Imperial College Press: London, **2014**.

that current is collected for a reasonably long time, the lower H_2O_2 concentration further away from the surface in the sensing experiments will reduce the current compared to that measured during calibration, in agreement with what was observed experimentally. For instance, to obtain a H_2O_2 concentration that is homogenous throughout the solution up to z = 1 mm, one would have to wait several days, while we only waited 5 min. During the chronoamperometry experiments, in which the potential is applied for ~30 s, H_2O_2 within a distance of more than $[2Dt]^{1/2} \approx 250$ µm will be able to diffuse to the electrode. C_{redox} is clearly significantly smaller than C_0 in this zone.

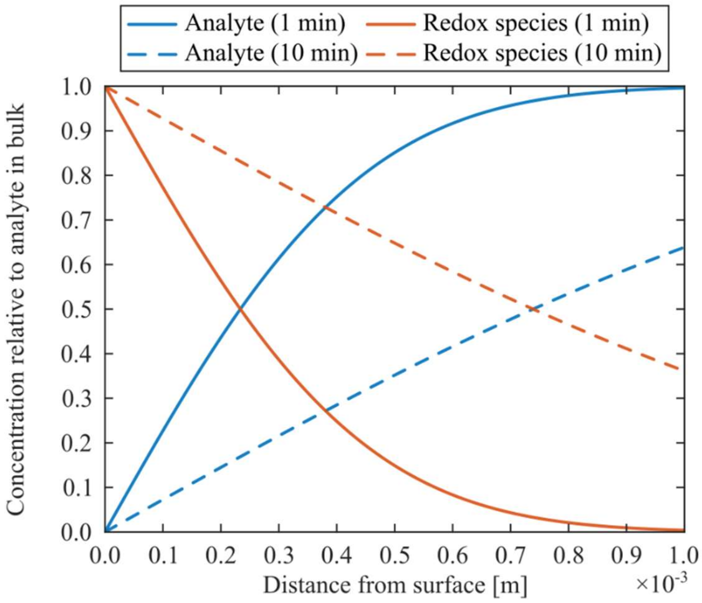

Figure S6 Concentration distribution of analyte (not redox active) and the redox active species generated by the enzymes at the interface ($z \approx 0$) after 1 min. The analyte is assumed to be converted to the redox species immediately when reaching the surface and the diffusion constants are assumed to be the same for both species. The liquid cell used in the experiments is even thicker than the maximum z value.

In conclusion, the lower charge transfer values for the neurotransmitter detection compared to the calibration can at least to a high extent be explained by the non-constant concentration profile in the former case.

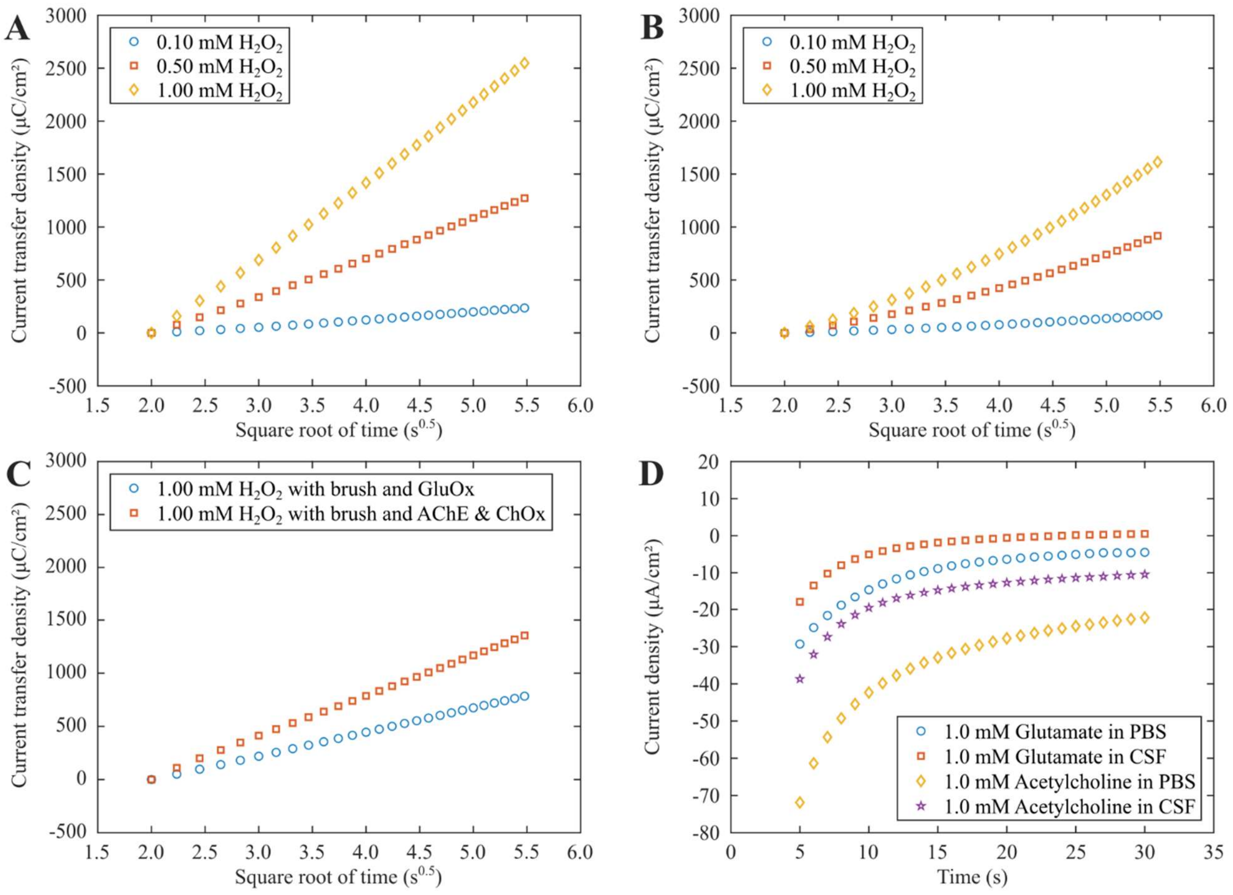

Figure S7 Additional chronoamperometry data. (A) Integrated response from exposing a bare gold surface to H_2O_2 at 0.10 mM, 0.50 mM and 1.00 mM concentrations. The response from pure PBS has been subtracted. (B) Integrated response (vs $t^{1/2}$) from exposing a surface with GOx brushes to bulk H_2O_2 concentrations of 0.10 mM, 0.50 mM and 1.00 mM. The relation is not linear, which is attributed to H_2O_2 interactions with GOx. (C) Integrated response (vs $t^{1/2}$) from exposing enzymatic brushes with GluOx or AChE + ChOx to a bulk H_2O_2 concentration of 1.00 mM. The relations are linear, in agreement with diffusion controlled redox reactions (Cottrell expression). (D) Amperometry curves recorded 10 min after exposing surfaces to 1.0 mM neurotransmitters, in PBS and in 10× diluted CSF.

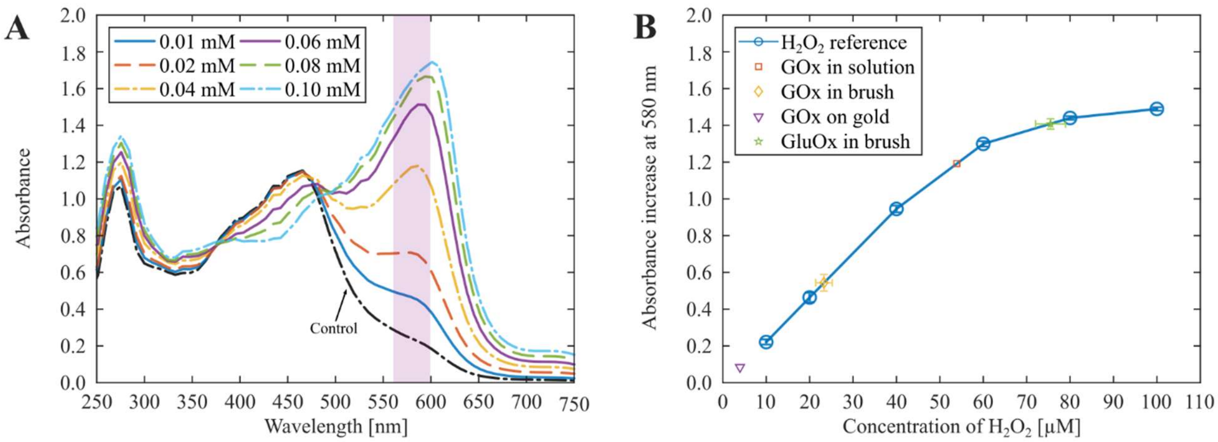

Figure S8 Colorimetric assay[3] (ferrous oxidation of xylenol orange) for independent detection of H_2O_2 generation and enzyme activity determination. (A) Spectra from calibration run with different H_2O_2 concentrations (zero as control). (B) Calibration curve and values measured for enzymatic breakdown reactions. For GOx in brush vs in solution, the absolute enzyme amounts were the same. GOx on gold means a directly adsorbed monolayer, for which the coverage was 190 ng/cm^2. Based on the sample are this gives a specific activity which is 51% of that for GOx in solution.

[3] Jiang, Z.-Y.; Woollard, A. C. S.; Wolff, S. P., Lipid hydroperoxide measurement by oxidation of Fe^{2+} in the presence of xylenol orange. Comparison with the TBA assay and an iodometric method. *Lipids* **1991**, 26, 853-856.

Substrate depletion

As mentioned in the main text, the specific activity for GOx in the brushes was ~50% lower compared to that of GOx in solution phase. This comparison was performed with the same amount of enzyme in both cases. Here we explain what we consider to be the most likely explanation, an effect we refer to as substrate depletion.

The reason for substrate depletion is simply diffusion in different geometries (Figure S9). GOx molecules in solution phase will be randomly distributed and evenly spaced from each other on average, while in the brush all GOx molecules are forced into extreme proximity. In terms of diffusion, the surface arrangement is the “worst possible” configuration because flux to a planar surface is lower than for any other geometry. (This is the reason why, for instance, microelectrodes give higher currents in voltammetry than larger ones.) In addition, the enzymes closest to the surface may also not encounter less glucose and/or oxygen, as these substrates are consumed by the enzymes located further away from the electrode surface. (It should be kept in mind that the enzymes are bound in multilayers to the brush.) In other words, enzymes deeper within the brush are likely capable of being fully active, but are not exposed to the same concentrations, which limits the rate. This issue does not arise in solution phase since mass transport can occur freely to individual enzymes in 3D (Figure S9).

We note that ordinary kinetic models (Michaelis-Menten) assume that mass transport plays no role. However, they also assume that the enzyme concentration is much lower than the substrate concentration. This is not the case inside our brushes, where the enzyme concentration is actually higher (> 1 mM) than the bulk substrate concentration (0.5 mM). This together with the planar surface geometry means that it can be expected that mass transport will play a role.

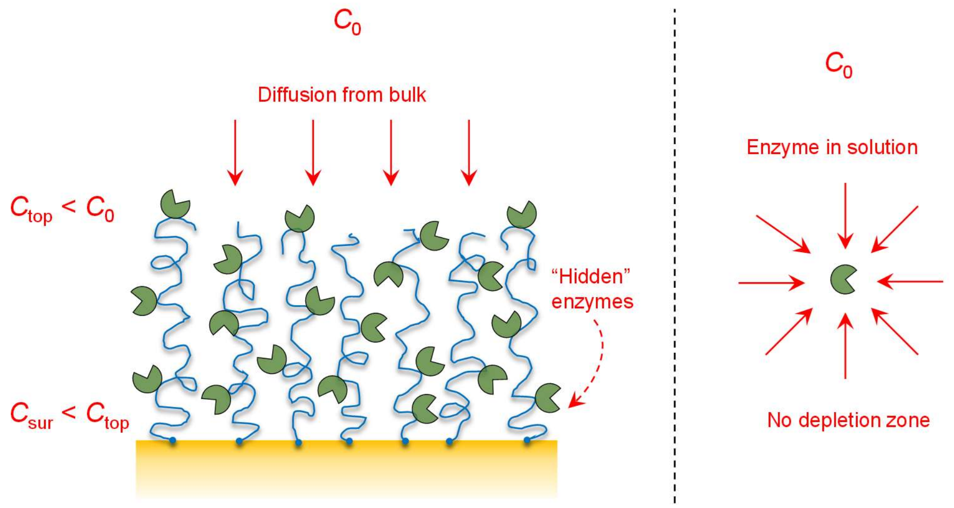

Figure S9 Illustration of the theory of limited substrate access. First, diffusion from the bulk might reduce the concentration at the top of the brush (C_{top}) in comparison with the bulk (C_0). Second, enzymes deep inside the brush may encounter less substrates (C_{sur}) than those on top of the brush. For a single enzyme free in solution, the diffusive transport is much more efficient and no depletion zone is expected, i.e. $C = C_0$ everywhere.

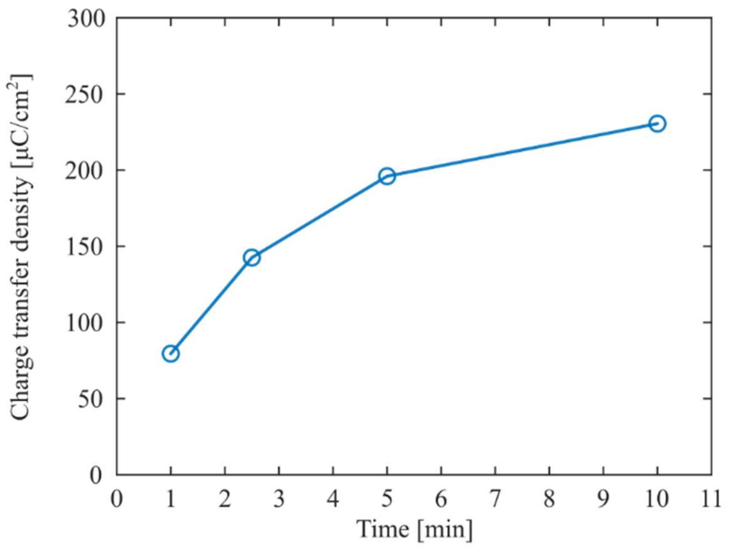

Figure S10 Example of time dependence of the signal from acetylcholine. The longer one waits after introducing the analyte, the larger the signal compared to the background. Note that this holds even though each measurement point consumes all H_2O_2 close to the surface.

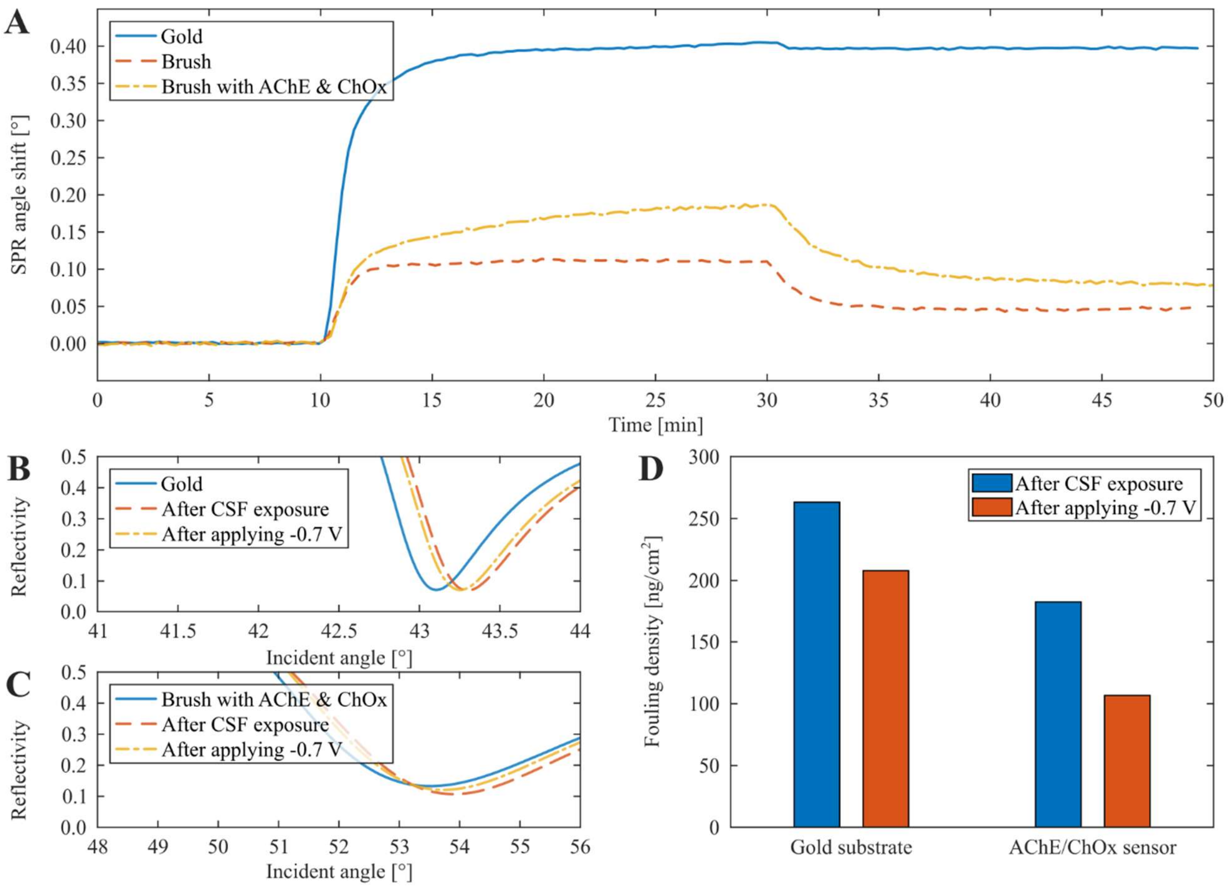

Figure S11 SPR data showing fouling from cerebrospinal fluid. (A) Real-time measurement during CSF injection (10× dilution) from 10 to 30 min. (B) SPR spectra in dry state for CSF on gold. (C) SPR spectra in dry state for an enzymatic brush. (D) Fouling amounts calculated based on the SPR data in dry state. The fouling on the enzymatic brush is less than on planar gold even though multilayers of molecules can fit inside the brush. When the potential is applied, some molecules are released as expected.[4]

[4] Ferrand-Drake del Castillo, G.; Kyriakidou, M.; Adali, Z.; Xiong, K.; Hailes, R. L. N.; Dahlin, A., Electrically switchable polymer brushes for protein capture and release in biological environments. *Angewandte Chemie International Edition* **2022**, 61, e202115745.

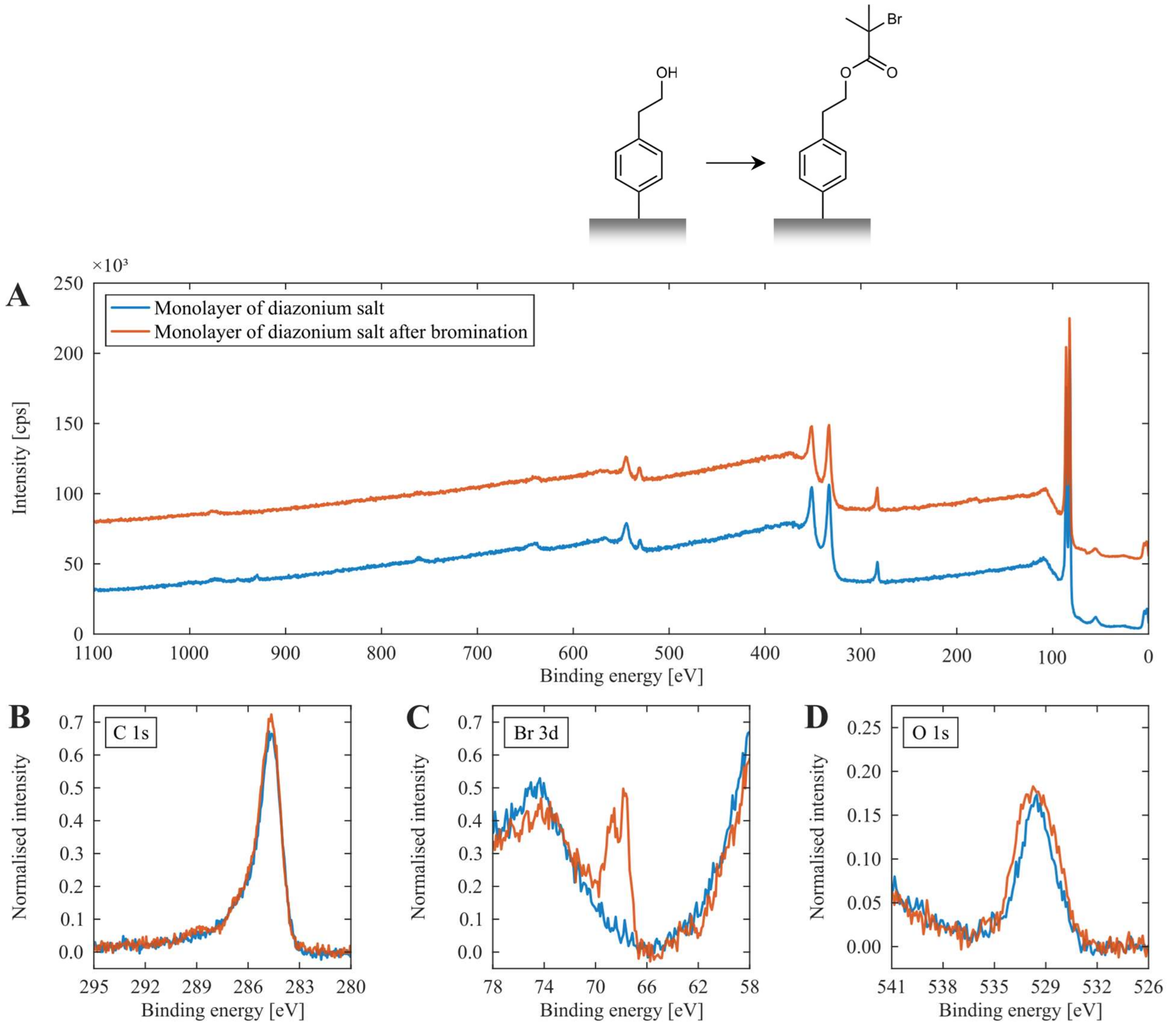

Figure S12 XPS spectra after diazonium salt grafting and after bromination. (A) Survey. (B) C 1s, no significant changes. (C) Br 3d, showing a peak only after bromination. (D) O 1s, slight peak broadening.

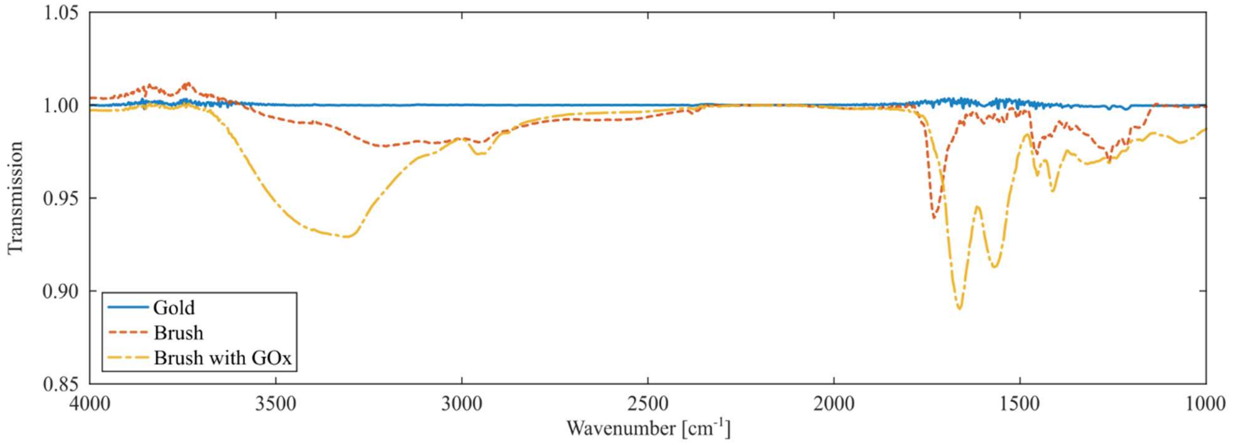

Figure S13 FTIR spectra of bare gold (solid), after polymer brush formation (dashed) and after ex-situ GOx immobilization (dotted). The spectrum of the polymer coating was similar to previously reported spectra of PAA,[5] with a wide band from 3600 cm^{-1} to 2500 cm^{-1} corresponding to stretching of the OH groups in the carboxylic acid and a peak centered on 1730 cm^{-1} corresponding to stretching of the C=O group in the carboxylic acid. After the immobilization of GOx these two were both diminished, which likely correlates to the EDC/NHS conversion and later conjugation with GOx. New peaks emerged at 1665 cm^{-1} and 1570 cm^{-1}, which is close to what has previously been shown to correlate to amide I and amide II bands,[6] and a band from 1440 cm^{-1} and 1270 cm^{-1}, which is close to what has previously been shown to correlate with carboxylate groups from the enzyme.[6] A new broad band also emerged from 3600 cm^{-1} to 3200 cm^{-1}, which usually corresponds to stretching of hydrogen bonded -OH groups and secondary amines on the surface, most likely from the enzymes.[7]

---

[5] Ferrand-Drake del Castillo, G.; Emilsson, G.; Dahlin, A., Quantitative analysis of thickness and pH actuation of weak polyelectrolyte brushes. *The Journal of Physical Chemistry C* **2018**, 122, 27516-27527.

[6] Kouassi, G. K.; Irudayaraj, J.; McCarty, G., Activity of glucose oxidase functionalized onto magnetic nanoparticles. *BioMagnetic Research and Technology* **2005**, 3, 1.

[7] Larkin, P., *Infrared and Raman spectroscopy: principles and spectral interpretation.* Elsevier: **2017**.

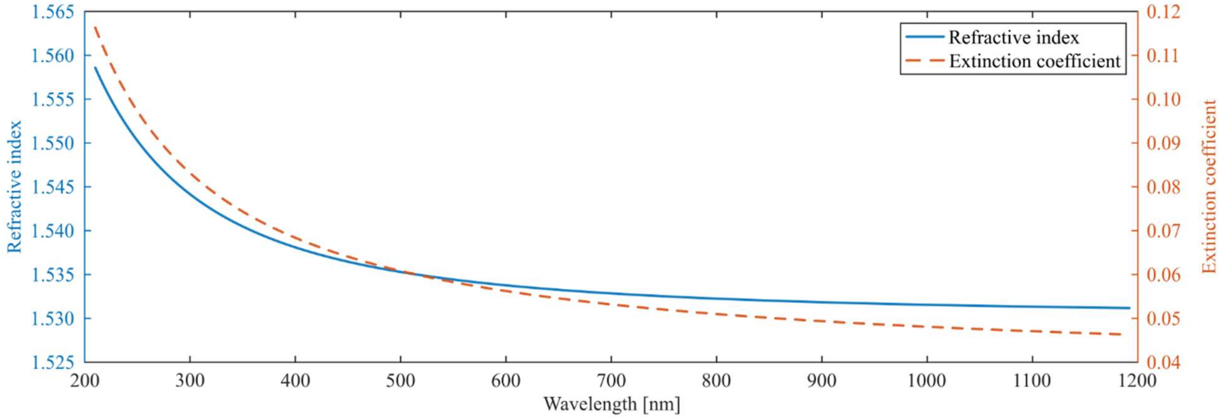

Figure S14 Spectroscopic ellipsometry analysis of a PAA brush. The data was obtained by first analyzing a reference gold surface. The thickness was determined to 9.1 nm and SPR on the same brush gave a thickness of 10.5 nm. This small discrepancy is mainly because the refractive index 1.527 was used for the SPR analysis, while ellipsometry shows 1.533 for the wavelength used.
